# Systematic review of defibrotide studies in the treatment of veno-occlusive disease/sinusoidal obstruction syndrome (VOD/SOS)

**DOI:** 10.1038/s41409-019-0474-8

**Published:** 2019-02-25

**Authors:** Paul Richardson, Saurabh Aggarwal, Ozlem Topaloglu, Kathleen F. Villa, Selim Corbacioglu

**Affiliations:** 1 Jerome Lipper Multiple Myeloma Center, Division of Hematologic Malignancy, Department of Medical Oncology, Dana-Farber Cancer Institute, Harvard Medical School, Boston, MA USA; 2 Novel Health Strategies, Bethesda, MD USA; 3 Jazz Pharmaceuticals, Inc, Palo Alto, CA USA; 4 Department of Pediatric Hematology, Oncology and Stem Cell Transplantation, University of Regensburg, Regensburg, Germany

**Keywords:** Drug therapy, Cancer

## Abstract

Veno-occlusive disease (VOD), also called sinusoidal obstruction syndrome (SOS), is a potentially life-threatening complication of hematopoietic stem cell transplantation (HSCT) conditioning or high-dose nontransplant chemotherapy. VOD/SOS with multi-organ dysfunction (MOD) is associated with a mortality rate of > 80%. Defibrotide (25 mg/kg/day) is approved to treat hepatic VOD/SOS with renal or pulmonary dysfunction post HSCT in the United States and to treat severe hepatic VOD/SOS in patients > 1 month of age in the European Union. A random effects model was used for pooling data from 17 systematically chosen defibrotide studies. For patients in these reports (*n* = 2598), and those in the subset of 10 reports of patients treated with ~ 25 mg/kg/day (*n* = 1691), estimated Day + 100 survival rates were 54% and 56%, respectively. Among those patients treated with ~ 25 mg/kg/day, estimated Day + 100 survival was 44% among patients with MOD and 71% in patients without MOD; survival was 41% and 70%, respectively, for the population of patients receiving any dose of defibrotide. Safety results were not pooled owing to differences in reporting methodology but were generally consistent with the known tolerability profile of defibrotide. This analysis provides the largest assessment of survival in patients treated with defibrotide for VOD/SOS with or without MOD.

## Introduction

Hepatic veno-occlusive disease (VOD), also called sinusoidal obstruction syndrome (SOS), is a potentially life-threatening complication of conditioning regimens for hematopoietic stem cell transplantation (HSCT) and also of chemotherapy alone [1–4]. Risk factors may be related to transplant (eg, the toxicity of chemotherapy or the conditioning regimen, allogeneic vs autologous transplant, immunosuppressive regimen), patient characteristics (eg, age, underlying disease, genetic predisposition), and health status of the liver (eg, immature liver function in infants, iron overload, liver fibrosis, hepatitis) [5, 6].

The pathogenesis of VOD/SOS involves multiple thrombotic and inflammatory factors that initially trigger damage to the endothelial cells lining the sinusoids of the hepatic acinus. Damaged endothelial cells may show cytopathic effects by rounding up, forming gaps in the sinusoidal barrier that allow passage of erythrocytes, leukocytes, and cellular debris into the space of Disse. As the venous lumen narrows and reduces the effluent from the sinus, post-sinusoidal portal hypertension occurs and can progress to the clinical symptoms of VOD/SOS [5–8].

Diagnosis of VOD/SOS has historically been based on clinical examination by either Baltimore criteria (bilirubin ≥ 2 mg/dL plus 2 or more of hepatomegaly, ascites, or ≥ 5% weight gain by Day 21 post HSCT) [9] or modified Seattle criteria (two or more of the following: bilirubin > 2 mg/dL, hepatomegaly or right upper quadrant pain, 2% weight gain [sometimes revised as 5% by Day 20 post HSCT [10]]) [11]. However, those criteria lack sensitivity and specificity, making early identification of VOD/SOS difficult. In addition, particularities in the presentation of VOD/SOD in children are not reflected in these diagnostic criteria. More recently, the European Society for Blood and Marrow transplantation (EBMT) has proposed new diagnostic guidelines and prospective severity grading criteria for adults and for children [7, 8]. The adult diagnostic criteria from EBMT encompass classical VOD/SOS as defined by Baltimore criteria but also include late-onset VOD/SOS (VOD/SOS developing after 21 days post HSCT). The new pediatric diagnostic criteria from EBMT include differences from the traditional criteria, such as having no defined timeframe of onset and the presence of unexplained consumptive/transfusion-refractory thrombocytopenia, otherwise unexplained weight gain for 3 consecutive days despite diuretic use, and rising bilirubin from baseline value on 3 consecutive days or bilirubin ≥ 2 mg/dL within 72 h. These new diagnostic guidelines are designed to support earlier diagnosis and treatment with greater specificity, and to highlight substantial differences in presentation between adult and pediatric patients (eg, anicteric presentation in ~ 30% of children which may be less common in adults presenting by Day 21 post HSCT) [7, 8].

VOD/SOS develops in ~ 10–15% of adult patients who receive myeloablative conditioning followed by allogeneic HSCT [1, 5, 12, 13]. In patients receiving autologous HSCT or reduced intensity conditioning with allogeneic HSCT, incidence may be ~ 5% [14], although a rate of 8.8% post–reduced intensity conditioning was reported in the past few years by one center [15]. Overall incidence in pediatric patients post HSCT has been reported between 22 and 30%, and in high-risk pediatric patients may increase to 60% [8]. The incidence in pediatric patients of VOD/SOS post-autologous HSCT for neuroblastoma is ~ 30%, likely owing to a busulfan–melphalan myeloablative conditioning [8].

VOD/SOS with multi-organ dysfunction (MOD; typically defined by renal and/or pulmonary dysfunction and sometimes referred to as multi-organ failure) may be associated with survival of 20–30% in HSCT patients receiving supportive care alone [1, 3, 4].

Owing to the progressive pathophysiology of VOD/SOS and the high mortality associated with VOD/SOS and MOD, the EBMT recommends that early diagnosis and treatment of VOD/SOS should be a priority, and they note that the “only proven” treatment is defibrotide [5]. For adult and pediatric patients, defibrotide (25 mg/kg/day intravenously in four divided doses) is approved to treat hepatic VOD/SOS with renal or pulmonary dysfunction post HSCT in the United States [16], and to treat severe hepatic VOD/SOS post HSCT in patients over 1 month of age in the European Union [17]. Defibrotide’s mechanism of action, as elucidated in preclinical studies, centers on protection of endothelial cells and anti-inflammatory effects, which together help restore thrombo-fibrinolytic balance [18–23].

To provide an estimate of overall survival in patients with VOD/SOS treated with defibrotide, we pooled systematically collected Day + 100 survival analysis data from published studies on the use of defibrotide to treat patients with VOD/SOS, post HSCT or post-nontransplant chemotherapy, with or without MOD.

## Materials and methods

### Search criteria

A systematic review of Medline and Medline In-Process for journal articles, and Embase for journal articles and conference abstracts, until July 10, 2017, was performed per a prespecified and clearly defined protocol based on Preferred Reporting Items for Systematic Reviews and Meta-Analyses (PRISMA) guidelines. Owing to the lag time of 3–6 months for conference abstracts in Embase, abstracts from the more recent 2017 EBMT and European Hematology Association meetings were also searched using the conference websites. The search term for all databases was “defibrotide” in the title or abstract. Duplicate results from these searches were removed.

### Criteria for study selection

Prospective and retrospective studies of defibrotide for treatment of VOD/SOS post HSCT or post-nontransplant chemotherapy were selected for inclusion. Excluded were reviews, prophylaxis or prevention studies, *post hoc* analyses, nonclinical studies, letters, clinical studies without primary efficacy data, no defibrotide treatment, and use in patients without VOD/SOS. Results from the initial keyword literature searches were screened, and full-length text for each report was evaluated for eligibility.

### Data collection

Full versions of the selected studies were assessed to determine study design, sample size, dose, treatment duration, patient characteristics (age, post-transplant or post-chemotherapy onset, and underlying disease), comparator(s), Day + 100 survival, and safety outcomes. When necessary, subgroup data were sourced from clinical study reports. For case series reporting patient-level data, the overall efficacy outcomes were estimated. Where reported, patients were divided into subgroups to analyze data for those with and without MOD, and for adult and pediatric patients.

### Biostatistical analysis

A random effects model was used for pooling data for survival. Interstudy heterogeneity was assessed with Cochran’s Q-test. The percentage of total variation across studies owing to heterogeneity was evaluated by the *I*^2^ measure. Owing to differences in defining and reporting adverse events (AEs) in the individual studies, pooling the data may have been misleading; therefore, safety was evaluated qualitatively. Pooled survival was estimated using StataSE software (StataCorp, College Station, TX).

## Results

**Fig. 1:**
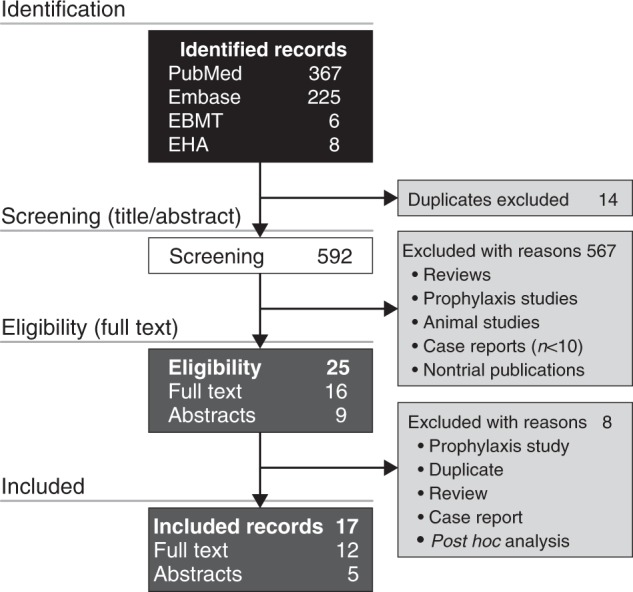
Study selection

The literature search based on the keyword “defibrotide” identified a total of 606 publications, which included 367 PubMed records, 225 Embase records, and 14 abstracts from the EBMT and European Hematology Association conferences (Fig. 1). Duplicates from these searches were removed.

After screening titles and abstracts for exclusion criteria, the remaining 25 complete records (16 full-text articles and nine conference abstracts) were analyzed and further refined by excluding eight reports that were *post hoc* analyses, reviews, duplicates, case reports, or a prevention trial. The 17 records chosen for pooled analysis were 12 full-length articles [3, 4, 24–33] and five abstracts [34–38] (Table 1). The study quality and types included retrospective case studies, single-center studies, registry reports, prospective multicenter compassionate use and treatment IND studies, and prospective multicenter two-arm phase 2 and 3 studies (Table 1). Patient ages ranged from 0.1 to 77 years, with median ages of 8.2–60.5 years. The combined studies included 2598 VOD/SOS patients treated with defibrotide, 1260 of whom had MOD (the precise definition of MOD varied among the studies). Most patients with VOD/SOS had received HSCT, and the most common primary diseases were acute leukemias [3, 4, 24, 26–33, 35, 37, 38] (Table 1). Defibrotide doses ranged from 5 mg/kg/day to 110 mg/kg/day, and the duration of defibrotide treatment ranged from 1–139 days with median duration ranging from 14–21.5 days (Table 2). Ten of the 17 reports included patients treated with approximately the approved 25 mg/kg/day defibrotide dose (*n* = 1691) [3, 25–27, 30, 34–38]. Seven of the 17 reports included other dosages, or the dosage was not reported [4, 24, 28, 29, 31–33].

**Table 1:** Summary of report characteristics, patient demographics, transplant type, and disease parameters

	Study quality factors			Defibrotide-treated			Age				Transplant		Common underlying diseases		
Reference	Prospective?	Type	Control	Total	MOD	No MOD	Median		≤ 16 years	> 16 years	Auto	Allo	ALL	AML	Other
Richardson 1998 [33]	Yes	Single center	No	19	19	0	40		3	16	11	8	1	3	15
Chopra 2000 [31]	Yes	CU	No	40	26	14	30		11	29	14	26	6	14	20
Richardson 2002 [28]	Yes	CU	No	88	88	0	35		29^a^	59^a^	28	60	6	22	60
Corbacioglu 2004 [29]	No	CU	No	45	22	23	8.2		40	5	8	37	6	10	29
Bulley 2007 [32]	No	Single center	No	14	NR	NR	10.2		14^b^	0^b^	0	14	3	1	10
Sucak 2007 [26]	No	Single center	No	14	6	8	40.5		0^b^	14^b^	1	13	4	5	5
Richardson 2010 [27]	Yes	Phase 2, dose finding	No	149	149	0	34		48^a^	101^a^	20	129	15	47	87
Ruiz Ramos 2014 [34]	No	Observational	No	11	NR	NR	NR		4	5	NR	NR	NR	NR	NR
Locatelli 2015 [35]	Yes	CU	No	98	17	77	13.4		52^a^	42^a^	10	75	18	23	57
Triplett 2015 [24]	Yes	Single center	No	34	22	12	8.9		31^a^	3^a^	2	29	10	10	14
Balade Martinez 2016 [36]	No	Observational	No	42	42	0	46		12	30	NR	NR	NR	NR	NR
Corbacioglu 2016 [30]	Yes	CU	No	710	261	348	25		303^a^	407^a^	112	499	120	177	413
Pol 2016 [25]	No	Single center	No	13	12	1	60.5		0	13	0	13	0	7	6
Richardson 2016 [3]	Yes	Phase 3	Historical	102	102	0	21		44	58	12	90	17	29	56
Strouse 2016 [4]	No	Registry	Not treated	41	41	0	11		25 (61)	16 (39)	2 (5)	39 (95)	19^c^		22
Yakushijin 2016 [37]	No	Registry	Thrombo-modulin	24	NR	NR	40		NR	NR	0	65	NR	NR	NR
Richardson 2017 (T-IND) [38]	Yes	T-IND	No	1154	571	NR	12		691	463	155	843	279	279	596

ALL, acute lymphocytic leukemia; Allo, allogeneic; AML, acute myelogenous leukemia; Auto, autologous; CU, compassionate use; MOD, multi-organ dysfunction; NR, not reported; T-IND, defibrotide expanded access program

^a^Pediatric defined as ≤ 18 years and adults as > 18 years

^b^Inclusive of age 16 years

^c^Acute leukemias

**Table 2:** Compilation of defibrotide treatment parameters

Reference	Total treated patients, *n*	Patients treated with ~ 25 mg/kg/d defibrotide, *n*	Median duration, days (range)	Defibrotide start time from VOD/SOS diagnosis	Mean dosage (mg/kg/d)	Median dosage (mg/kg/d)	Dosage range (mg/kg/d)
Richardson 1998 [33]	19	NR	NR	Median 6 days (range, 0–47 days)	NR	NR	5–60
Chopra 2000 [31]	40	NR	18 (2–71)	NR	NR	NR	10–40
Richardson 2002 [28]	88	NR	15 (1–139)	Median 3 days (range, 0–46 days)	NR	NR	5–60
Corbacioglu 2004 [29]	45	NR	17 (1–83)	Median 1 day (range, 0–12 days)	45 in the CR group; 27 in the no responder group	40	10–110
Bulley 2007 [32]	14	NR	16 (4–37)	Median 1 day (range, 0–33 days)	25 (starting dosage)	33–38.5	11–81
Sucak 2007 [26]	14	14	21.5 (4–39)	As soon as possible	NR	NR	10–25
Richardson 2010 [27]	149	75	19 (2–82)^a^	Day of randomization (*n* = 119 [80%])	25^a^	25^a^	25, 40
Ruiz Ramos 2014 [34]	11	11	9 (5–25)	NR	NR	25	25–40
Locatelli 2015 [35]	98	94	14 (1–84)	NR	NR	25	6.15–40.0
Triplett 2015 [24]	34	NR	15 (1–102)	Median 0 days	NR	60	6.25–110
Balade Martinez 2016 [36]	42	42	11 (1–40)	NR	25	NR	10–45
Corbacioglu 2016 [30]	710	227	15 (1–119)	NR	NR	25	10–80
Pol 2016 [25]	13	13	14 (6–21)	Within 24 h	25	25	25
Richardson 2016 [3]	102^b^	102^b^	21.5 (1–58)	NR	25	25	25
Strouse 2016 [4]	41	NR	NR	NR	NR	NR	NR
Yakushijin 2016 [37]	24	24	15 (1–46)	NR	NR	24^c^	7–80^c^
Richardson 2017 (T-IND) [38]	1154	1089	21	NR	25	25	25

CI, confidence interval; CR, complete responder; NR, not reported; VOD/SOS, veno-occlusive disease/sinusoidal obstruction syndrome

^a^Among the 75 patients who received defibrotide 25 mg/kg/d (treatment arm A)

^b^Among the 102 patients who received defibrotide 25 mg/kg/d in the active treatment group (not including the 32 historical controls)

^c^Median dose or dose range (mg/kg)

### Efficacy

**Fig. 2:**
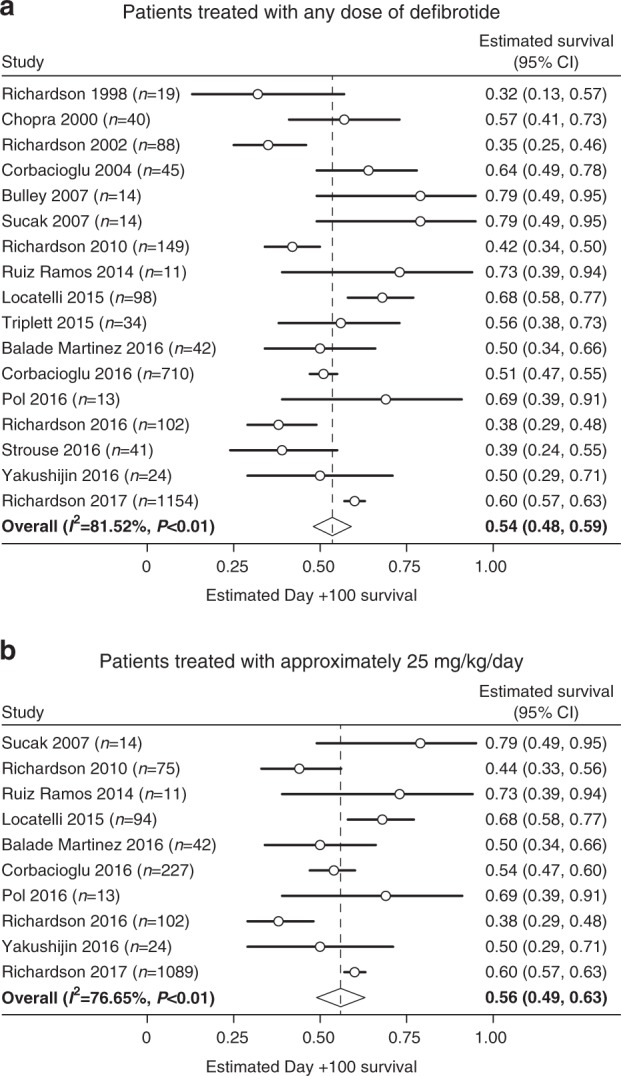
Pooled analysis of the estimated Day + 100 survival rates of the overall patient populations treated with any defibrotide dose or ~ 25 mg/kg/day

The estimated Day + 100 survival rate for 2598 patients receiving defibrotide at any dose across all studies [3, 4, 24–38] was 54% (Fig. 2a), and a 56% rate was shown among the 1691 patients in 10 reports of treatment at ~25 mg/kg/day (Fig. 2b) [3, 25–27, 30, 34–38].

**Fig. 3:**
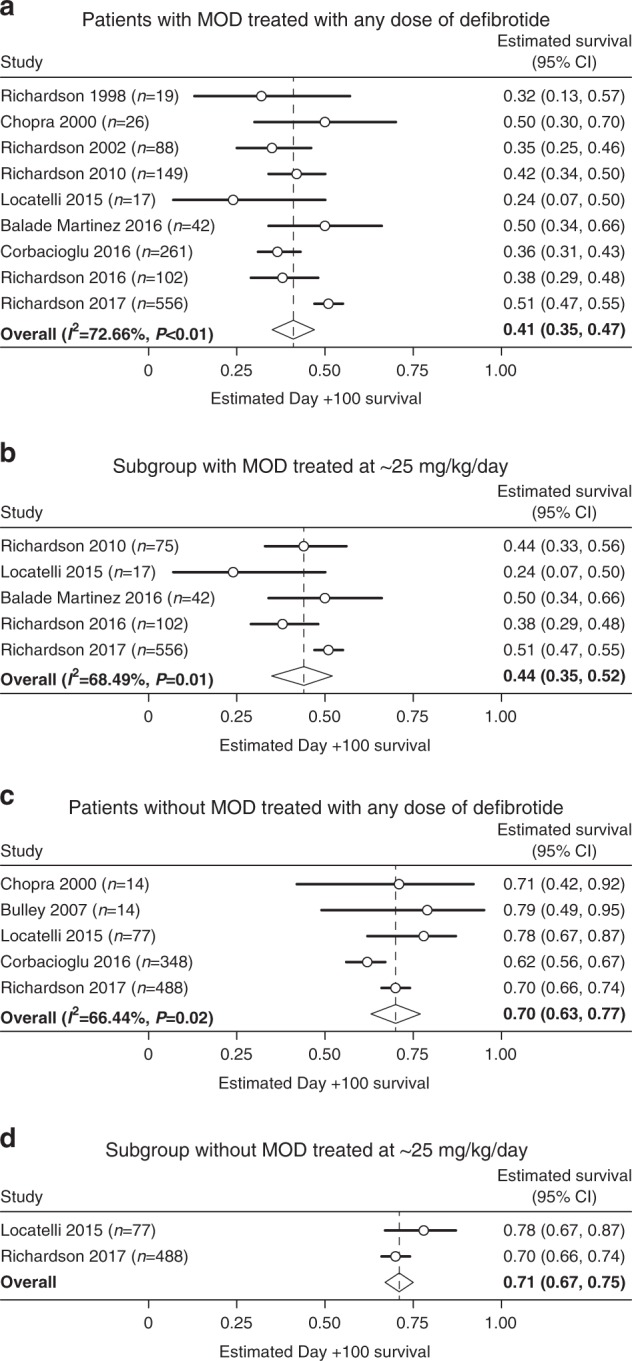
Estimated Day + 100 survival for patients with MOD and without MOD

Pooled subgroup results showed that patients with MOD (*n* = 1260) who received any of the defibrotide doses [3, 27, 28, 30, 31, 33, 35, 36, 38] had an estimated Day + 100 survival rate of 41% (Fig. 3a), and a 44% rate was shown in the ~ 25 mg/kg/day subgroup (*n* = 792; Fig. 3b) [3, 27, 35, 36, 38].

Among the subgroup of patients without MOD (*n* = 941) receiving defibrotide at any dose [30–32, 35, 38], estimated Day + 100 survival was 70% for those receiving any dose (Fig. 3c), and 71% in those receiving ~ 25 mg/kg/day (*n* = 565; Fig. 3d) [35, 38].

**Fig. 4:**
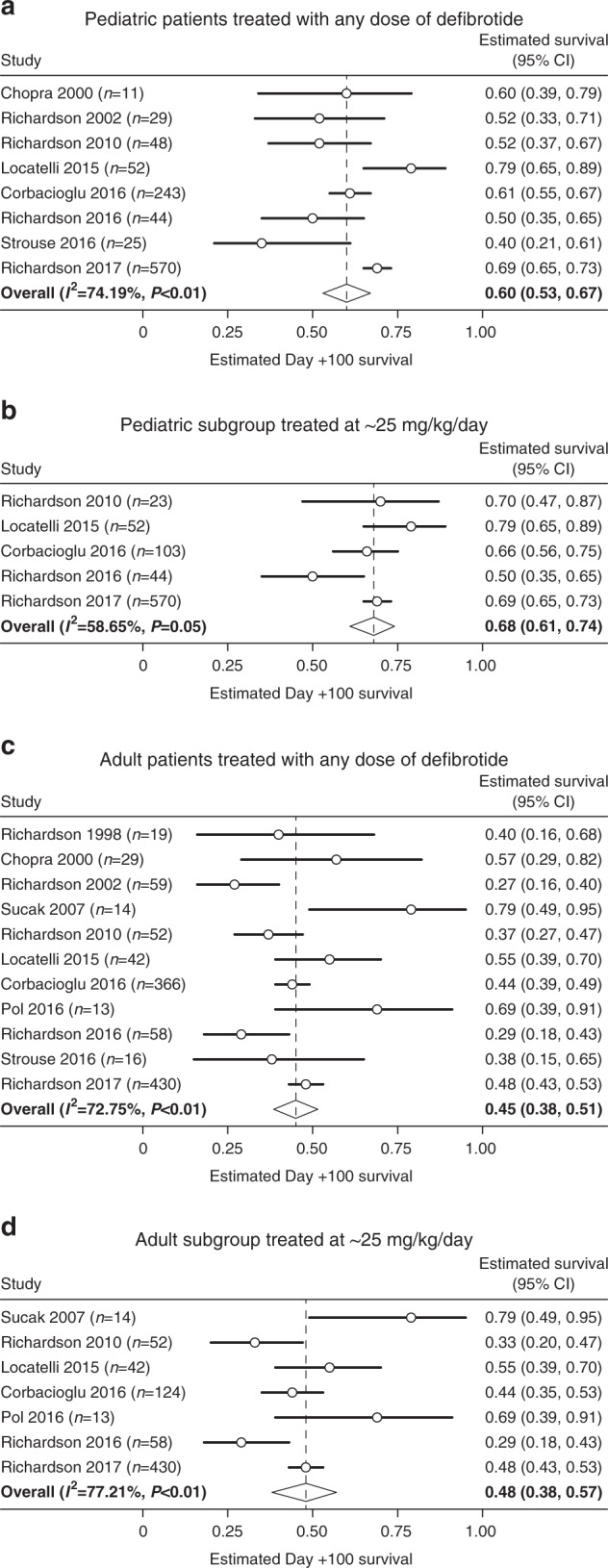
Estimated Day + 100 survival rates in pediatric and adult subgroups

The pediatric subgroup was defined as patients aged ≤ 16 years in three studies [3, 4, 38] and ≤ 18 years in five studies [27, 28, 30, 31, 35]. Pediatric patients with VOD/SOS, regardless of MOD status and dose (*n* = 1036) [3, 4, 27, 28, 30, 31, 35, 38] had an estimated Day + 100 survival rate of 60% (Fig. 4a), whereas the subgroup that received ~ 25 mg/kg/day dose (*n* = 792) [3, 27, 30, 35, 38] had a 68% estimated Day + 100 survival rate (Fig. 4b). Three of the 25 mg/kg/day studies included patients with MOD, with pooled Day + 100 survival of 58% (95% CI: 51–66%) [3, 27, 38].

Adults were defined as patients aged > 16 years in six studies [3, 4, 25, 26, 35, 38] and as > 18 years in five studies [27, 28, 30, 31, 33]. The adult subgroup, regardless of MOD status and dose (*n* = 1128) [3, 4, 25–28, 31, 33, 35, 38, 39] had an estimated Day + 100 survival rate of 45% (Fig. 4c), whereas the subgroup that received the defibrotide dose of ~ 25 mg/kg/day (*n* = 773) [3, 25–27, 30, 35, 38] had an estimated Day + 100 survival rate of 48% (Fig. 4d). Three of the 25 mg/kg/day studies included patients with MOD, with pooled Day + 100 survival of 36% (95% CI: 29–42%) [3, 27, 38].

Safety results for the included reports are summarized in Table 3. Safety results were not pooled for these studies owing to differences in safety reporting methodology; however, the results of individual studies were generally consistent with the previously reported safety profiles, such as in the phase 3 historically controlled trial in VOD/SOS patients with MOD [3]. That trial reported that 101/102 defibrotide-treated patients and all 32 historical control patients experienced ≥ 1 adverse event (AE). Hypotension was the most frequent AE (39% for defibrotide, 50% for controls), and common hemorrhagic AEs, which included pulmonary alveolar and gastrointestinal hemorrhage, occurred in 64% of defibrotide-treated patients and 75% of controls. Treatment-related AEs in the defibrotide arm included hemorrhagic events and hypotension [3].

**Table 3:** Adverse event summaries extracted from the published articles

Reference	Total treated patients	Adverse events	Treatment-related AEs
Richardson 1998 [33]	19	Grade 1/2 transient mild systolic hypotension (*n* = 5 [26%]) and grade 3/4 hypotension (*n* = 2 [11%]); but causal relationship not reported	None caused defibrotide discontinuation. No severe treatment-related hemorrhage was reported
Chopra 2000 [31]	40	Not reported	Not reported
Richardson 2002 [28]	88	No worsening of clinical bleeding was seen. No other grade 3/4 toxicity related to DF was reported. Grade 1/2 AEs included transient mild systolic hypotension (treatment attribution not reported)	No grade 3/4 treatment-related AEs were reported
Corbacioglu 2004 [29]	45	Not reported	1 patient discontinued DF owing to diarrhea. Mild (grades 0–2) coagulation abnormalities (*n* = 16 [36%]); none led to discontinuation
Bulley 2007 [32]	14	GI bleed (*n* = 2 [14%]; 1 patient had GI bleed 1 day prior to initiation of defibrotide) and intracranial bleed (*n* = 1 [7%]; likely owing to disseminated fungal infection)	Not reported
Sucak 2007 [26]	14	Mild-to-moderate AEs included abdominal pain (*n* = 4 [28.6%]) and mild mucosal bleeding (*n* = 5 [35.7%]); none led to discontinuation	No significant drug-related toxicities. As patients were thrombocytopenic, it was not clear whether mild mucosal bleeding events were related to defibrotide)
Richardson 2010 [27]	149	71/75 (95%; treatment arm A [25 mg/kg/d defibrotide]) and 73/74 (99%; treatment arm B [40 mg/kg/d defibrotide]) patients reported ≥ 1 treatment-emergent AE	Grades 3–5 AEs were in arm A (5/75, 7%) and arm B patients (7/74, 10%). Discontinuations from TRAE were owing to hypotension in 1 patient in each arm ( ≤ 1.4%); 1 grade 3–5 bleeding event in arm B; and alveolar bleeding (*n* = 1 [1.4%] in arm A) and GI and pulmonary bleedings (*n* = 1 [1.3%] each in arm B)
Ruiz Ramos 2014 [34]	11	3 (27.3%) patients with hemorrhagic episodes (2 GI and 1 nasal)	Not reported
Locatelli 2015 [35]	98	21 (21%) patients with ≥ 1 treatment-emergent AE; most common ( ≥ 2 patients) were MOD (9%), VOD/SOS (6%), and CMV infection and acute respiratory distress syndrome (2% each)	5 (5.3%) patients had hemorrhagic events. 1 patient each had hemorrhagic cystitis, urinary tract hemorrhage, hemorrhages (unspecified), hemorrhagic diathesis, and pulmonary hemorrhage
Triplett 2015 [24]	34	Patients receiving ≤ 100 mg/kg/day had average of 3 bleeding episodes/100 days; 13.2 bleeding episodes/100 days in patients receiving > 100 mg/kg/d	Not reported
Balade Martinez 2016 [36]	42	Not reported	Not reported
Corbacioglu 2016 [30]	710	53% of patients reported an AE. The most common AEs ( ≥ 4%) included new or worsening MOD, progression of hepatic VOD/SOS, sepsis, and GVHD	The relationship to defibrotide was not available for the majority of AEs
Pol 2016 [25]	13	Not reported	Not reported
Richardson 2016 [3]	102	101/102 (99%; DF treatment group) and 32/32 (100%; historical controls) patients experienced ≥ 1 AE. Incidence of common hemorrhagic AEs (pulmonary alveolar [11.8% and 15.6%] and GI [7.8% and 9.4%]) was similar between defibrotide and control groups, respectively	11 (10.7%) patients discontinued defibrotide owing to a possible drug-related toxicity
Strouse 2016 [4]	41	Not reported	Not reported
Yakushijin 2016 [37]	24	Not reported	1 (4%) patient experienced 2 treatment-related AEs (GI and pulmonary bleeding)
Richardson 2017 (T-IND) [38]	1154	810 (70.2%) patients reported ≥ 1 treatment-emergent AE	248 (21.5%) patients; led to discontinuation in 12% and death in 2.7% (pulmonary hemorrhage, 1.0%, was most common). Hypotension in 2.1% patients, and hemorrhagic events in pulmonary (4.3%), GI (3.0%), and epistaxis (2.3%)

AEs, adverse events; ALL, acute lymphocytic leukemia; CMV, cytomegalovirus; DF, defibrotide; GI, gastrointestinal; GVHD, graft-vs-host disease; MOD, multi-organ dysfunction; T-IND, defibrotide expanded access program; TRAE, treatment-related adverse event; VOD/SOS, veno-occlusive disease/sinusoidal obstruction syndrome

## Discussion

This systematic, pooled analysis of currently available evidence for defibrotide efficacy in the treatment of patients with VOD/SOS included 17 studies, representing 2598 patients. Estimated Day + 100 survival rates in the 17 studies ranged from 35 to 79%, and the pooled survival rate at Day + 100 was 54%. The approved 25 mg/kg/day dose was administered to patients in 10 of 17 studies (*n* = 1691), and its estimated survival rate at Day + 100 was 56%. This pooled analysis further supports the efficacy found for the 25 mg/kg/day dose of defibrotide in the phase 3 study [3] that was approved by regulatory authorities.

Patients with VOD/SOS and MOD, regardless of treatment dose, had lower estimated survival rates at Day + 100 than those without MOD: estimated survival rates for patients treated with any defibrotide dose or treated with the ~ 25 mg/kg/day defibrotide dose were 41% and 44%, respectively. As points of comparison, Day + 100 survival in the historical control population (rigorously selected owing to ethical concerns regarding withholding a supposed beneficial treatment from very sick patients with a dismal prognosis) in the phase 3 study was 25% [3], and in prior reports, patients with MOD who did not receive defibrotide were shown to have Day + 100 survival results of 30.9% [4] and 15.7% [1]. Conversely, the estimated survival rates at Day + 100 for patients without MOD were higher than those for the overall population: 70% for patients treated with any dose and 71% for those treated with ~ 25 mg/kg/day. Of note, the definition of MOD varied among studies, and the large sponsored studies used a standard that represented the most severe forms (renal dysfunction typically defined by creatinine ≥ 3 × level at time of transplant or creatinine clearance/glomerular filtration rate ≤ 40% of baseline, or dialysis dependence; pulmonary dysfunction typically defined by oxygen saturation ≤ 90% on room air or need for supplemental oxygen/ventilator dependence [3, 38]).

Estimated survival rates at Day + 100 for pediatric patients with and without MOD, treated with any defibrotide dose or treated with the approximately 25 mg/kg/day defibrotide dose were 60% and 68%, respectively, with 58% survival in the MOD subgroup receiving 25 mg/kg/day; in the T-IND study, survival at Day + 100 for pediatric patients with MOD was 58.1% [40]. In comparison, the US registry included in the pooled analysis also reported Day + 100 survival among patients not receiving defibrotide to be 45.5% among pediatric patients with MOD [4].

Overall, estimated Day + 100 survival rates for adults were 45% for those who received any dose and 48% for those receiving ~25 mg/kg/day, with 36% survival in the MOD subgroup receiving 25 mg/kg/day; in the T-IND study, survival at Day + 100 for adults with MOD was 39.0% [41]. In comparison, a Japanese registry of primarily adult patients (84.2% aged ≥ 16 years), 95% of whom did not receive defibrotide, had a Day + 100 survival rate of 32% in patients with and without MOD, and a rate of 15% in the MOD subgroup [13]. In the US registry, Day + 100 survival was 27.3% among adults with MOD not receiving defibrotide [4].

The AE reports for the 17 studies could not be pooled across studies owing to distinct reporting schemes. However, the overall AE profiles in the 17 studies were similar to those reported in the defibrotide phase 3 study [3]. In that study, AEs assessed by investigators as at least possibly related to defibrotide included hemorrhagic events and hypotension. Of note, however, overall rates of hypotension and hemorrhage (regardless of relatedness) were similar between arms. Hypotension was the most frequently reported AE (39% for defibrotide, 50% for controls), and hemorrhagic AEs, which included pulmonary alveolar hemorrhage (11.8% vs 15.6%, respectively) and gastrointestinal hemorrhage (7.8% vs 9.4%, respectively), occurred in 64% of defibrotide-treated patients and 75% of controls [3].

Safety results from the two large single-arm studies, both of which included patients without MOD, found no novel AEs [30, 38]. Final results from the T-IND showed an AE rate of 70.2%, with serious AEs reported in 51.8%, whereas AEs considered treatment-related were most commonly hemorrhage (pulmonary, 4.3%; gastrointestinal, 3.0%; epistaxis, 2.3%) and hypotension (2.0%); serious treatment-related AEs occurred in 11.5% of patients [38]. In the compassionate use program, data reporting was not required owing to the nature of the study [30]. AEs were reported in 53% of patients, and causes of death (frequently reported as AEs) were primarily owing to progressive VOD/SOS with MOD.

A key strength of this analysis is that it represents the largest, most comprehensive review of Day + 100 survival in patients with VOD/SOS who were treated with defibrotide. In most cases, however, patient-level data were not available to control for heterogeneity between studies, including differences in baseline characteristics, such as severity of MOD (eg, reduced pulmonary/renal function vs ventilator/dialysis dependence), which represents a limitation in that it was not possible to retrospectively apply the new severity criteria proposed by EBMT or to pool safety data. Another consideration in interpretation of these results is that the largest reports were from single-arm studies designed to provide access to defibrotide [30, 38]; however, the estimated Day + 100 survival results are comparable to those of the phase 3 historically controlled trial (in patients with MOD only, with a propensity-adjusted number-needed-to-treat of five to prevent one death) [3, 42, 43], and the safety profile in the phase 3 study helps illustrate the range of AEs associated with VOD/SOS and MOD irrespective of treatment with defibrotide.

The results in the patients without MOD may be supportive of treatment earlier in the pathophysiologic cascade of VOD/SOS. An exploratory analysis from the T-IND on the impact of timing of initiation with defibrotide on outcome found that earlier treatment initiation was associated with improved survival [44], which is consistent with what is known about the pathophysiologic cascade of VOD/SOS progression and the treatment recommendations from the EBMT [5]. Although mortality of VOD/SOS without prospectively identified MOD has not been well studied, a Japanese registry reported that VOD/SOS without MOD was associated with Day + 100 mortality > 50% [13]. Indeed, prevention of development of both MOD and VOD/SOS itself are important areas for research. At present, no medications are approved for the prevention of VOD/SOS, although defibrotide has been investigated in several studies [45–47] including a phase 3 study in pediatric patients, which suggest that defibrotide may reduce the risk for development of VOD/SOS compared with supportive care only (12% vs 20% incidence, respectively; *P* = 0.0488, competing risk analysis, *P* = 0.0507 log rank test) [48]. Hemorrhage was the AE most commonly attributed by investigators to defibrotide; however, the incidence was similar between groups: 22% in the defibrotide arm and 21% in the control arm [49]. The HARMONY clinical trial (NCT02851407) to compare efficacy and safety of defibrotide versus best supportive care in the prevention of VOD/SOS in pediatric and adult patients is continuing to recruit patients [49]. Also, awareness of the importance of early intervention for improved outcomes may lead to a shift in the application of diagnostic criteria, from emphasis on the more exclusionary Baltimore and Seattle criteria to the more age-specific fit of the EBMT criteria. Finally, early use of magnetic resonance imaging for evaluating iron overload and/or ultrasound imaging to confirm such clinical criteria as ascites or hepatomegaly for VOD/SOS diagnosis and intervention may be of benefit to high-risk patients [5]. Elastographic methods also are under investigation to detect early markers of VOD/SOS, which may lead to earlier diagnosis and treatment [50].

In this pooled analysis of studies of defibrotide given approximately at the approved dose of 25 mg/kg/day for the treatment of VOD/SOS, estimated Day + 100 survival was 56% in the 2073 patients with or without MOD. As expected, survival in patients with VOD/SOS without MOD was greater at Day + 100 (69%) than in patients with VOD/SOS with MOD (42%). Safety results in the individual studies were generally consistent with the known safety profile of defibrotide. Taken together, these results show a largely consistent treatment effect for defibrotide in the broad population of patients treated with defibrotide for VOD/SOS, with or without MOD.
